# m^6^A RNA Methylation-miRNA Crosstalk in Cardiovascular Remodeling

**DOI:** 10.3390/biom16060858

**Published:** 2026-06-11

**Authors:** Liujie Long, Yi Yang, Chufang Zheng, Kang Kang

**Affiliations:** Department of Biochemistry and Molecular Biology, Shenzhen University Medical School, Shenzhen 518060, China; 2023221151@email.szu.edu.cn (L.L.); yy@szu.edu.cn (Y.Y.); 2022221008@email.szu.edu.cn (C.Z.)

**Keywords:** cardiovascular disease, vascular remodeling, non-coding RNAs, m^6^A, epitranscriptomics, extracellular vesicles, endothelial cells, vascular smooth muscle cells, fibroblasts, cardiomyocytes

## Abstract

Cardiovascular remodeling, encompassing vascular remodeling, myocardial remodeling, and fibrosis-associated tissue remodeling, underlies atherosclerosis, pulmonary hypertension, myocardial infarction, myocardial fibrosis, and other cardiovascular diseases. Its regulation has traditionally been studied through transcriptional, inflammatory, metabolic, mechanical, and intercellular signaling mechanisms. Recent advances in epitranscriptomics have identified N6-methyladenosine (m^6^A) RNA methylation as an additional post-transcriptional layer that interacts with microRNA (miRNA) pathways during cardiovascular disease progression. This review summarizes current evidence for m^6^A-miRNA crosstalk in cardiovascular remodeling, focusing on epitranscriptomic checkpoints that regulate miRNA fate, feedback-like regulatory circuits involving miRNAs and the m^6^A machinery, and cell-type-specific programs across endothelial cells, vascular smooth muscle cells, fibroblasts, and cardiomyocytes. We further discuss emerging analytical technologies and translational implications of this regulatory axis. Future studies should clarify causal mechanisms, cell-type and disease-stage specificity, and translational feasibility. Together, this multilayered framework provides a systems-level perspective on how RNA regulatory networks may shape pathological remodeling in cardiovascular disease.

## 1. Introduction

Cardiovascular remodeling is a broad pathological process underlying a wide spectrum of cardiovascular and pulmonary vascular diseases, including atherosclerosis, hypertension, restenosis, pulmonary hypertension, myocardial infarction, cardiac hypertrophy, and heart failure [1,2,3,4]. Although these conditions affect distinct anatomical compartments, they share common remodeling features, including vessel wall reorganization, myocardial injury, fibroblast activation, extracellular matrix remodeling, inflammation, and persistent changes in tissue structure and function. Accordingly, this review uses cardiovascular remodeling as an integrated framework encompassing vascular remodeling, myocardial remodeling, and fibrosis-associated tissue remodeling. Within this framework, vascular remodeling refers to vessel-centered changes involving endothelial cells, vascular smooth muscle cells, vascular fibroblasts, and pulmonary vascular lesions, whereas myocardial or cardiac remodeling refers to cardiomyocyte injury, infarction-associated remodeling, cardiac hypertrophy, and cardiac fibrosis. These processes integrate mechanical stress, inflammatory signaling, hypoxia, metabolic perturbations, and intercellular communication, highlighting the need to understand how dynamic pathological cues are linked to durable remodeling-associated cell states [5,6,7,8,9,10].

MicroRNAs (miRNAs) have emerged as key post-transcriptional regulators of cardiovascular remodeling [11,12,13,14]. By simultaneously targeting multiple mRNAs, miRNAs orchestrate gene expression programs that govern cell proliferation, migration, phenotypic switching, inflammatory activation, and cell survival. In vascular smooth muscle cells, for example, miRNAs regulate the transition between contractile and synthetic states [15,16], whereas in endothelial cells, they modulate angiogenesis and barrier function [17]. In cardiomyocytes and fibroblasts, miRNA-mediated regulation also contributes to injury responses, regeneration, and fibrotic remodeling [18,19]. However, changes in miRNA abundance alone cannot fully explain the context specificity and durability of remodeling-associated phenotypes, suggesting the existence of additional regulatory layers controlling miRNA biogenesis and activity.

RNA methylation, particularly N6-methyladenosine (m^6^A)—dynamically regulated by “writers” (e.g., methyltransferase-like 3 and 14 (METTL3 and METTL14), WT1-associated protein (WTAP)), “erasers” (e.g., fat mass and obesity-associated protein (FTO), demethylase AlkB homolog 5 (ALKBH5)), and “readers” (e.g., YTH N6-methyladenosine RNA-binding protein family (YTHDF), YTH domain-containing protein family (YTHDC), heterogeneous nuclear ribonucleoprotein A2/B1 (hnRNPA2B1))—has recently emerged as a major epitranscriptomic mechanism that regulates RNA fate at multiple levels, including splicing, stability, translation, and decay [20,21]. In cardiovascular systems, dysregulation of RNA methylation has been implicated in diverse pathological processes, such as cardiac hypertrophy, ischemic injury, fibrosis, and pulmonary vascular remodeling [22,23,24]. Notably, seminal studies demonstrate that m^6^A modification directly promotes primary miRNA processing through recognition by DiGeorge syndrome critical region 8 (DGCR8) and associated RNA-binding proteins (RBPs) [25,26], while nuclear readers such as hnRNPA2B1 further facilitate miRNA maturation in an m^6^A-dependent manner [27]. These findings establish a direct mechanistic link between RNA methylation and miRNA biogenesis, providing a potential explanation for how miRNA programs acquire context specificity.

Previous reviews have summarized these advances from the perspectives of miRNA-mediated regulation in cardiovascular remodeling, m^6^A RNA methylation in cardiovascular disease, and broader interactions between RNA modifications and non-coding RNAs. However, RNA methylation and miRNA regulation have often been discussed as related but separate regulatory layers, and the specific logic by which m^6^A-dependent miRNA regulation is organized across the miRNA lifecycle, reciprocal regulatory circuits, and cardiovascular cell types remains insufficiently synthesized. For example, one review has cataloged bidirectional interactions between m^6^A modifications and non-coding RNAs in oncological contexts [28]; however, these interactions have not been conceptualized as hierarchically organized regulatory systems governing cell-state transitions in cardiovascular remodeling. Building on this bidirectional interaction, we propose that m^6^A RNA methylation-miRNA crosstalk constitutes a regulatory axis that may help coordinate remodeling-associated cell-state transitions during cardiovascular remodeling.

To structure this conceptual framework, this review is organized around three interconnected regulatory levels: epitranscriptomic checkpoints governing miRNA fate, feedback-like regulatory circuits between miRNAs and the m^6^A machinery, and cell-type-specific programs that contribute to cardiovascular remodeling. We therefore synthesize current knowledge on RNA methylation-mediated control of the miRNA lifecycle, miRNA-dependent regulation of epitranscriptomic machinery, and their integrated roles across endothelial, vascular smooth muscle, fibroblast, and cardiomyocyte populations.

The literature search was performed in PubMed and was last updated in April 2026. Search terms combined RNA methylation-related keywords, including “RNA methylation”, “RNA modification”, “epitranscriptome”, “epitranscriptomic”, “epitranscriptomics”, “m^6^A”, “N6-methyladenosine”, METTL3, METTL14, WTAP, FTO, ALKBH5, YTHDF, YTHDC, IGF2BP, hnRNPA2B1, and related sequencing methods, with terms related to miRNAs and cardiovascular remodeling-associated cell types, processes, and diseases. Priority was given to original studies and recent reviews directly addressing RNA methylation-mediated miRNA regulation, miRNA-dependent regulation of RNA methylation machinery, or related mechanisms in cardiovascular and cardiovascular-relevant models. Non-cardiovascular fibrotic studies were included selectively when they provided mechanistic insight into conserved fibroblast activation or extracellular matrix remodeling relevant to cardiovascular fibrosis.

## 2. miRNA Biogenesis Under Epitranscriptomic Control

**Figure 1:**
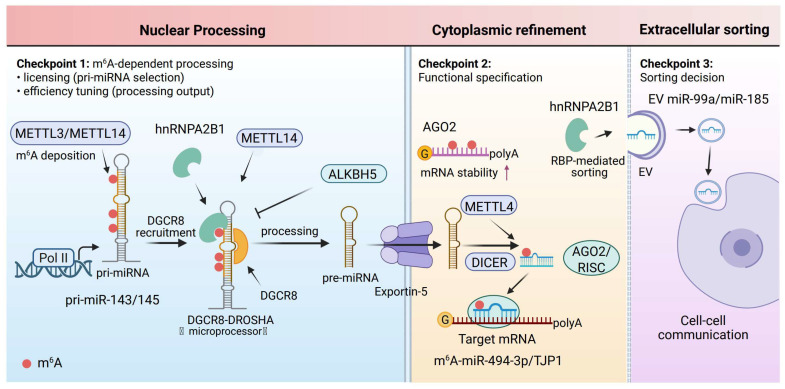
Epitranscriptomic checkpoints governing miRNA biogenesis and fate. m^6^A RNA methylation-miRNA crosstalk is organized through a series of stage-specific regulatory checkpoints spanning the miRNA lifecycle. In the nucleus, m^6^A deposition on pri-miRNAs establishes a processing checkpoint that licenses selected pri-miRNAs for microprocessor engagement and dynamically tunes processing efficiency, thereby determining mature miRNA output. Representative examples include m^6^A-dependent processing of pri-miR-143/145. In the cytoplasm, m^6^A further refines miRNA function by modulating target recognition, mRNA stability, and Argonaute 2 (AGO2)/RNA-induced silencing complex (RISC)-mediated silencing, as illustrated by m^6^A-modified miR-494-3p-mediated repression of tight junction protein 1 (TJP1). Finally, selective loading of miRNAs into extracellular vesicles is mediated by RNA-binding proteins such as hnRNPA2B1, defining a terminal checkpoint that governs miRNA sorting and intercellular communication, as exemplified by EV-associated miR-99a and miR-185. Together, these checkpoints define a regulatory framework through which m^6^A methylation coordinates miRNA fate from biogenesis to functional deployment and intercellular transfer. Created in BioRender. Kang, K. (2026) https://BioRender.com/ktuo7uf, accessed on 5 June 2026. BioRender, Toronto, ON, Canada.

Accumulating evidence indicates that m^6^A modification dynamically regulates miRNA biogenesis across multiple stages, ranging from primary transcript processing to mature miRNA function and extracellular sorting [29]. Rather than representing a strictly linear cascade, miRNA maturation is governed by a series of stage-specific epitranscriptomic checkpoints that collectively determine whether pri-miRNAs are processed, how efficiently they are matured, and how mature miRNAs are functionally deployed or selectively exported (Figure 1).

### 2.1. Epitranscriptomic Control of Pri-miRNA Processing

An early step in miRNA biogenesis is governed by m^6^A-dependent regulation of pri-miRNA processing [30,31]. METTL3-mediated m^6^A deposition marks pri-miRNAs and promotes their recognition by DGCR8, thereby enabling microprocessor-mediated cleavage, whereas depletion of METTL3 reduces DGCR8 binding and leads to accumulation of unprocessed pri-miRNAs [32]. These findings establish m^6^A as a permissive signal that determines whether pri-miRNAs are selected for processing, thereby defining an epitranscriptomic licensing checkpoint at the entry of the miRNA biogenesis pathway.

Beyond this initial selection step, the same m^6^A-dependent mechanism is dynamically modulated to fine-tune processing output. The nuclear m^6^A reader hnRNPA2B1 binds m^6^A-marked pri-miRNAs and facilitates their interaction with DGCR8, thereby promoting processing efficiency [27,33]. Similarly, METTL14 enhances DGCR8-mediated processing through m^6^A-dependent mechanisms, whereas ALKBH5 antagonizes this process by removing m^6^A marks and impairing DGCR8 recruitment [31,34,35]. For example, disruption of m^6^A signaling impairs processing of pri-miRNAs such as miR-143/145 in pulmonary artery smooth muscle cells [36], illustrating the functional consequences of perturbed m^6^A-dependent processing. These observations indicate that pri-miRNA processing is not a binary event but is continuously tuned, defining a processing checkpoint that integrates both entry selection and efficiency control to determine mature miRNA abundance.

### 2.2. Cytoplasmic Refinement of miRNA Activity

Following nuclear processing, RNA methylation further regulates miRNA function at the cytoplasmic stage, representing a distinct layer of post-processing control over miRNA activity. Mature miRNAs can harbor m^6^A modifications that directly influence target repression, as demonstrated by studies showing context- and site-dependent changes in repression efficacy toward downstream targets [37]. In endothelial cells, site-specific m^6^A-modified miR-494-3p, but not its unmethylated form, is required for repression of tight junction protein 1, highlighting that RNA methylation can determine target specificity [38]. Beyond direct modification of miRNAs themselves, m^6^A also regulates components of the miRNA effector machinery. For example, m^6^A modification of *AGO2* mRNA regulates its stability, thereby influencing global miRNA-mediated silencing capacity [39]. These findings define a functional checkpoint at which RNA methylation refines miRNA output by shaping target recognition and silencing efficiency, thereby fine-tuning post-transcriptional gene regulation.

### 2.3. miRNA Secretion and Extracellular Communication

Beyond intracellular activity, specific miRNAs are selectively sorted into extracellular vesicles (EVs) rather than being passively released [40,41]. This process can be mediated by RNA-binding proteins such as hnRNPA2B1, which recognize defined sequence motifs and guide miRNAs into vesicular pathways [42]. Experimental studies demonstrate that hnRNPA2B1-dependent loading governs intercellular transfer of miRNAs such as miR-99a and miR-185, thereby modulating recipient cell function [43,44]. These findings indicate that extracellular export of miRNAs is a regulated process, defining a terminal checkpoint that determines whether mature miRNAs are retained intracellularly or exported for intercellular communication.

Collectively, these findings support a checkpoint-based framework in which RNA methylation regulates miRNA fate across nuclear processing, functional specification, and extracellular sorting, providing a structured framework for understanding how upstream signals may be linked to stage-specific control of miRNA activity.

## 3. Feedback Loops Between miRNAs and the RNA Methylation Machinery

**Figure 2:**
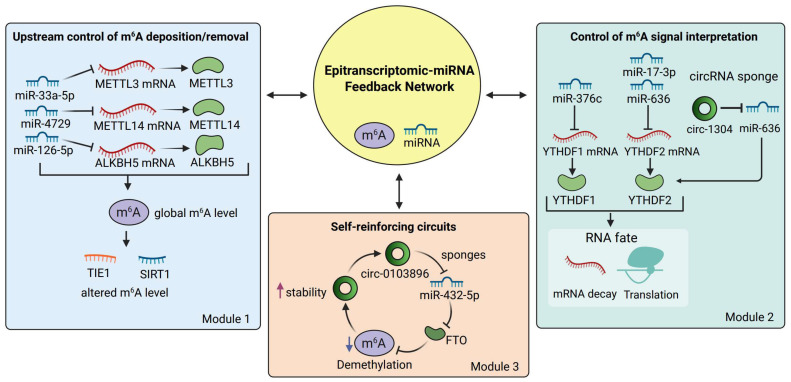
Feedback loops between the m^6^A machinery and miRNAs. Bidirectional interactions between m^6^A regulation and miRNAs form a dynamic regulatory network that integrates upstream control, m^6^A signal interpretation, and feedback circuit formation. Within this framework, miRNAs regulate m^6^A writers and erasers, reshaping the RNA methylation landscape (module 1), and target reader proteins to modulate the interpretation of m^6^A-modified transcripts (module 2). Representative axes illustrate the direction of regulation from miRNAs to m6A writers, erasers, and readers, including miR-33a-5p/METTL3, miR-4729/METTL14, miR-126-5p/ALKBH5, miR-376c/YTHDF1, and circ-1304/miR-636/YTHDF2. In certain contexts, these interactions form self-reinforcing circuits involving circular RNAs, miRNAs, and m^6^A regulators, as illustrated by the circ_0103896/miR-432-5p/FTO feedback loop (module 3). Together, these examples illustrate how feedback-like regulatory architectures may contribute to dynamic and context-dependent regulation. Created in BioRender. Kang, K. (2026) https://BioRender.com/ktuo7uf, accessed on 5 June 2026. BioRender, Toronto, ON, Canada.

m^6^A-miRNA crosstalk in cardiovascular cells is not merely unidirectional but instead operates through layered and reciprocal regulatory interactions that adapt to disease-specific microenvironments. These interactions encompass upstream control of epitranscriptomic machinery, modulation of m^6^A signal interpretation, and the formation of self-reinforcing feedback circuits (Figure 2).

### 3.1. miRNAs Targeting m^6^A Writers and Erasers

miRNAs can directly regulate the m^6^A machinery by targeting core writers and erasers, thereby reshaping RNA methylation dynamics at the upstream regulatory level. In vascular smooth muscle cells, miR-33a-5p suppresses METTL3 expression and attenuates oxidized low-density lipoprotein (ox-LDL)-induced calcification [45]. Similarly, miR-4729 targets METTL14 in endothelial cells, reducing global m^6^A levels and weakening tyrosine kinase with immunoglobulin-like and EGF-like domains 1 (TIE1)-dependent angiogenic signaling [46]. Beyond writers, erasers are also subject to miRNA-mediated control. For example, miR-126-5p modulates ALKBH5-dependent m^6^A regulation of sirtuin 1 (SIRT1), thereby influencing endothelial survival and angiogenic responses [47]. Together, rather than acting solely as downstream effectors, these miRNAs function as upstream modulators of the epitranscriptomic machinery, dynamically tuning RNA methylation states in response to cellular cues.

### 3.2. miRNAs Targeting m^6^A Readers

At a distinct regulatory layer, miRNAs modulate the interpretation of m^6^A signals by regulating reader proteins. Current cardiovascular evidence is strongest for YTH N6-methyladenosine RNA-binding protein 1 (YTHDF1) and YTH N6-methyladenosine RNA-binding protein 2 (YTHDF2). In pulmonary hypertension, the circALMS1/miR-17-3p/YTHDF2 axis has been reported to regulate hypoxia-induced pulmonary microvascular endothelial cell dysfunction, linking reader regulation to endothelial injury and vascular remodeling [48]. In neointimal hyperplasia, bioinformatic prediction followed by dual-luciferase reporter assays showed that miR-636 targets YTHDF2, whereas circ-1304 sequesters miR-636 and increases YTHDF2 abundance in vascular smooth muscle cells [49]. Functionally, YTHDF2 promotes degradation of m^6^A-modified mechanistic target of rapamycin (*mTOR*) mRNA, activates vascular smooth muscle cell (VSMC) autophagy, and exacerbates neointimal hyperplasia. Similarly, in pregnancy-induced hypertension, luciferase reporter assays confirmed the interaction between miR-19b-3p and YTHDF1; circYTHDF1-mediated sequestration of miR-19b-3p increases YTHDF1 expression and promotes endothelial ferroptosis and apoptosis [50]. Compared with YTHDF1/2, direct miRNA-mediated regulation of other readers remains less well characterized in cardiovascular remodeling, although miR-let7 has been reported to target insulin-like growth factor 2 mRNA-binding protein 1 (IGF2BP1) in atherosclerosis-associated models [51]. Direct miRNA regulation of YTH domain-containing protein 1 (YTHDC1) or YTH domain-containing protein 2 (YTHDC2) remains poorly documented. Together, these findings suggest that reader-directed miRNA regulation is an emerging layer of m^6^A-miRNA crosstalk that influences RNA decay, translation, autophagy, endothelial injury, and vascular remodeling.

### 3.3. Epitranscriptomic Feedback Circuits

Beyond linear regulatory interactions, certain epitranscriptomic-miRNA axes may form reciprocal circuits that help sustain RNA regulatory states. In intracranial aneurysm, circ_0103896 functions as a sponge for miR-432-5p, thereby increasing FTO expression in vascular smooth muscle cells [52]. In the same study, FTO was reported to directly interact with circ_0103896, reduce its m^6^A modification, and increase circ_0103896 RNA abundance, thereby forming a positive circRNA-miRNA-eraser feedback loop. Although the authors interpreted these findings as FTO-mediated stabilization of circ_0103896, the evidence was mainly based on FTO-dependent changes in circ_0103896 m^6^A enrichment, RNA abundance, and FTO-circ_0103896 interaction, rather than direct RNA decay or half-life assays.

This feedback module can also be interpreted within a broader RNA regulatory network. Other cardiovascular studies have identified FTO-centered competing endogenous RNA (ceRNA) or circular RNA (circRNA)-m^6^A modules. For example, hsa_circ_0005255 acts through the hsa-miR-3916/FTO/m^6^A axis in chronic hypoxia-related myocardial injury, whereas circCELF1 integrates miR-636/dickkopf Wnt signaling pathway inhibitor 2 (DKK2) regulation with FTO-dependent m^6^A control in myocardial fibrosis [18,53]. These examples suggest that FTO-associated RNA regulatory modules may be shaped by multiple upstream non-coding RNAs, m^6^A-dependent mechanisms, and RNA-binding interactions. However, whether these modules operate as self-reinforcing feedback circuits remains to be defined.

## 4. m^6^A-miRNA Circuits in Cardiovascular Cells

**Figure 3:**
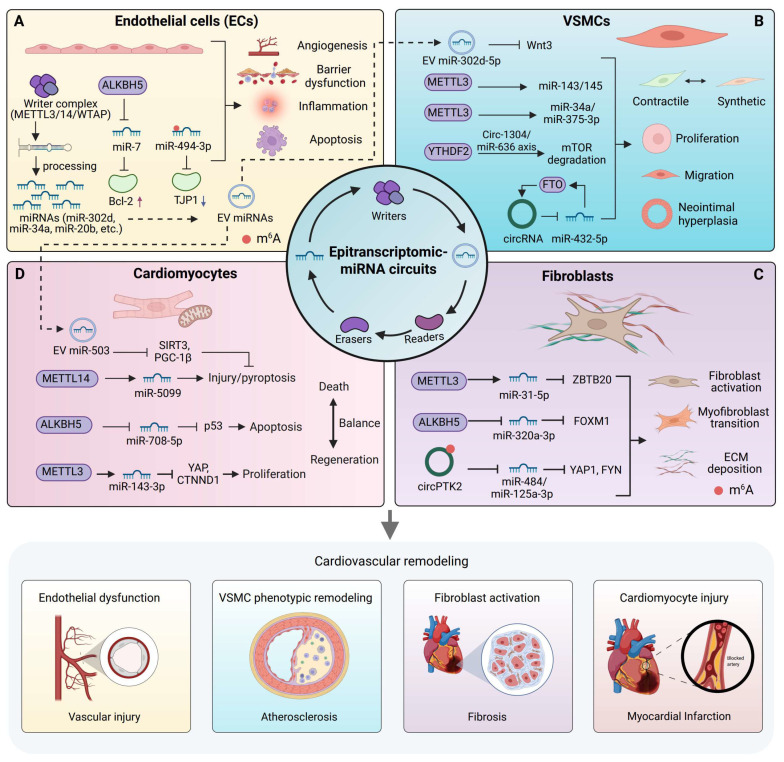
Cell-type-specific m^6^A-miRNA circuits in cardiovascular remodeling. m^6^A-miRNA crosstalk provides an integrated regulatory framework that operates across cardiovascular cell types while producing distinct functional outcomes. The central module illustrates the integration of m^6^A writers, erasers, and readers with miRNA networks and EV-mediated communication, forming interconnected regulatory circuits that coordinate miRNA biogenesis and activity. Representative axes, directions of regulation, and associated phenotypic consequences are shown for each cell type. (**A**) In endothelial cells, m^6^A-dependent regulation of pri-miRNA processing, mature miRNA function, and EV-mediated transfer modulates key processes such as angiogenesis, barrier integrity, inflammation, and apoptosis. (**B**) In VSMCs, these circuits govern phenotypic plasticity by regulating the transition between contractile and synthetic states, ultimately influencing proliferation, migration, and neointimal remodeling. (**C**) In fibroblasts, m^6^A-miRNA interactions contribute to activation programs, myofibroblast transition, and extracellular matrix deposition, thereby promoting fibrotic remodeling. (**D**) In cardiomyocytes, these circuits regulate cell fate decisions by balancing pyroptosis, apoptosis, mitochondrial dysfunction, and regenerative responses following injury, thereby influencing cardiac remodeling. At the systems level, cell-type-specific dysregulation of these circuits may converge to promote cardiovascular remodeling, which in turn gives rise to key pathological outcomes, including vascular injury, atherosclerosis, fibrosis, and myocardial infarction. Created in BioRender. Kang, K. (2026) https://BioRender.com/ktuo7uf, accessed on 5 June 2026. BioRender, Toronto, ON, Canada.

A central unresolved question is how a common set of m^6^A-miRNA molecular components gives rise to distinct cell-type-specific functional outputs during cardiovascular remodeling. This section examines how epitranscriptomic checkpoints and feedback loops are deployed in a lineage-specific manner, generating diverse remodeling responses across the four major cardiovascular cell types—endothelial cells, vascular smooth muscle cells, fibroblasts, and cardiomyocytes (Figure 3). Representative m^6^A-miRNA regulatory circuits are summarized in Table 1.

### 4.1. Endothelial Cells

Epitranscriptomic regulation of miRNA programs has emerged as an important contributor to endothelial plasticity during vascular remodeling. A recurring feature is that m^6^A-dependent control of pri-miRNA processing acts as an upstream regulatory layer that influences endothelial responses toward either adaptive or pathological trajectories.

At the level of miRNA biogenesis, in vascular calcification and aging, melatonin enhances WTAP-dependent m^6^A deposition on pri-miR-302d, promoting its maturation and exosomal transfer from endothelial cells to vascular smooth muscle cells. In recipient cells, this miRNA suppresses Wnt3 signaling and limits calcific and senescent transformation [57]. Similarly, in preeclampsia-associated endothelial injury, METTL14-driven methylation of pri-miR-34a facilitates its processing, leading to forkhead box protein P1 (FOXP1) repression and exacerbation of endothelial apoptosis and inflammation [63]. In contrast, angiogenic competence also relies on METTL3 activity: by sustaining the production of let-7e-5p and miR-18a-5p, METTL3 suppresses thrombospondin-1 and promotes adaptive neovascularization [54]. Under hypoxia/reoxygenation stress, METTL3-dependent maturation of miR-20b restrains excessive autophagy via unc-51-like autophagy activating kinase 1 (ULK1) targeting, thereby protecting endothelial cells from injury [56]. By contrast, eraser-mediated regulation counterbalances these effects. ALKBH5 suppresses pri-miR-7 maturation, thereby maintaining B-cell lymphoma 2 (Bcl-2) expression and protecting endothelial cells from tumor necrosis factor alpha (TNFα)-induced apoptosis [61]. Together, these findings position m^6^A writers and erasers as functionally opposing rheostats that fine-tune endothelial responses during vascular remodeling.

Beyond unidirectional control, feedback regulation further stabilizes and reinforces these programs. Reduced miR-4729 expression relieves repression of METTL14, increasing global and TIE1-specific m^6^A modification and activating pro-angiogenic signaling [46]. In contrast, delivery of miR-126-5p via engineered extracellular vesicles suppresses ALKBH5, enhances m^6^A modification of SIRT1, and preserves endothelial viability under fibrotic stress [47]. These examples indicate that miRNAs not only act downstream of methylation but actively reshape the epitranscriptomic landscape through reciprocal regulation. Representative miRNA-m^6^A machinery interactions are summarized in Table 2.

Regulation also extends to the level of mature miRNA function. Site-specific m^6^A modification of miR-494-3p enhances its ability to repress tight junction protein 1 (TJP1), thereby compromising endothelial barrier integrity and accelerating intracranial atherosclerosis [38]. Thus, RNA methylation can modulate target specificity independently of miRNA abundance, adding a post-maturation layer of regulatory precision.

Finally, integration with non-coding RNA networks introduces an additional layer of regulatory complexity. Insulin-like growth factor 2 mRNA-binding protein 2 (IGF2BP2) stabilizes m^6^A-modified long intergenic non-protein-coding RNA 1116 (LINC01116), enabling sequestration of miR-210-3p and promoting angiogenic signaling [64]. Similarly, reduced METTL3-mediated m^6^A modification stabilizes metastasis-associated lung adenocarcinoma transcript 1 (MALAT1), amplifying the MALAT1/miR-23a-3p/vascular endothelial growth factor A (VEGFA) axis and driving endothelial dysfunction in diabetic retinopathy [65]. Through these reader-long non-coding RNA (lncRNA)-miRNA circuits, RNA methylation coordinates transcript stability with competitive endogenous RNA regulation to fine-tune endothelial signaling outputs.

Collectively, these findings support a multilayered regulatory framework in which m^6^A-dependent control of miRNA biogenesis, activity, and network integration contributes to endothelial adaptation and injury during vascular remodeling.

### 4.2. Vascular Smooth Muscle Cells

m^6^A-miRNA crosstalk has been increasingly implicated in phenotypic switching of vascular smooth muscle cells (VSMCs), a key driver of vascular remodeling across pulmonary hypertension, atherosclerosis, aneurysm formation, and neointimal hyperplasia. In contrast to endothelial cells, where regulation often reflects adaptive responses, epitranscriptomic control in VSMCs frequently determines the stability of contractile versus synthetic cell states.

A central mechanism involves m^6^A-dependent regulation of miRNA biogenesis that modulates phenotypic programs. In pulmonary hypertension, METTL3-mediated m^6^A deposition facilitates the processing of pri-miR-143/145 through hnRNPA2B1, sustaining expression of these contractile miRNAs and restraining phenotypic switching. Disruption of this pathway reduces miR-143/145 levels, leading to upregulation of Krüppel-like factor 4 (KLF4) and suppression of contractile gene expression [36]. Similarly, in atherosclerosis, METTL3 promotes maturation of miR-375-3p via DGCR8-dependent processing, thereby regulating the 3-phosphoinositide-dependent protein kinase-1 (PDK1) pathway and facilitating VSMC phenotypic transformation [59]. In abdominal aortic aneurysm, METTL3-driven m^6^A modification enhances pri-miR-34a processing, leading to SIRT1 repression and exacerbation of disease progression [58]. Together, these findings indicate that m^6^A-dependent miRNA maturation functions as a regulatory node that shapes VSMC phenotypic states toward either contractile stability or pathological remodeling.

Beyond writer-dependent control of miRNA maturation, m^6^A signals are further integrated through reader- and eraser-associated regulatory circuits. In neointimal hyperplasia, the circ-1304/miR-636/YTHDF2 axis links miRNA activity to mRNA stability and autophagy, thereby promoting vascular remodeling [49]. Conversely, in intracranial aneurysm the circ_0103896/miR-432-5p/FTO feedback loop limits smooth muscle phenotypic switching and attenuates disease progression [52]. These examples highlight that epitranscriptomic-miRNA circuits extend beyond linear regulation to form interconnected networks that reinforce or constrain VSMC phenotypic states in a context-dependent manner.

Overall, these findings support the view that m^6^A-miRNA crosstalk acts as an important regulatory axis contributing to VSMC phenotypic state transitions during vascular remodeling.

### 4.3. Fibroblasts and Matrix-Producing Cells

In fibroblasts, m^6^A-miRNA crosstalk contributes to fibroblast activation, myofibroblast differentiation, and extracellular matrix deposition, which are central processes in cardiovascular fibrosis and adverse tissue remodeling [66]. Compared with endothelial cells, VSMCs, and cardiomyocytes, direct evidence linking m^6^A-miRNA regulation to cardiovascular fibroblast biology remains relatively limited. Therefore, selected non-cardiovascular fibrotic models are discussed here only as supportive mechanistic analogs, not as direct cardiovascular disease evidence. These examples are included to illustrate conserved principles of fibroblast activation, myofibroblast transition, and matrix remodeling that may be relevant to cardiac and vascular fibrosis.

A central mechanism involves m^6^A-dependent control of miRNA biogenesis that governs fibroblast activation in cardiovascular fibrotic remodeling. In myocardial fibrosis, epitranscriptomic-miRNA circuits have been shown to couple m^6^A-dependent regulation with miRNA-mediated target repression, thereby modulating fibroblast activation and extracellular matrix production [18]. This finding establishes a direct link between m^6^A-dependent RNA regulation and fibroblast-driven cardiac remodeling.

Consistent with these mechanisms, related regulatory principles are observed in other fibrotic contexts with relevance to cardiovascular disease. In pulmonary fibrosis, ALKBH5 exerts an opposing effect by demethylating pri-miR-320a, thereby preventing DGCR8-mediated processing and reducing mature miRNA levels, which leads to enhanced forkhead box M1 (FOXM1) expression and fibroblast activation [31]. In arsenic-induced pulmonary fibrosis, METTL3-dependent m^6^A modification of lncRNA E230001N04Rik regulates miR-20b-3p and promotes epithelial cell senescence, thereby inducing myofibroblast differentiation through intercellular signaling [67]. These findings highlight that RNA methylation-miRNA crosstalk can operate across cell types to amplify fibrotic responses.

More broadly, mechanistic insights from non-cardiovascular fibrotic models further support the generalizability of this regulatory axis. For example, METTL3-mediated m^6^A modification of pri-miR-31 drives fibroblast activation and collagen deposition in hypertrophic scar formation [68], underscoring a conserved epitranscriptomic mechanism of fibroblast activation.

Regulation further expands through circRNA-mediated and reader-associated networks, particularly in cardiovascular fibrotic remodeling. In myocardial fibrosis, the circCELF1/miR-636/DKK2 (dickkopf Wnt signaling pathway inhibitor 2) axis modulates fibroblast activation by coupling FTO-dependent m^6^A regulation with miRNA-mediated target repression [18]. More recently, m^6^A-modified circRNAs such as circPTK2 have been shown to drive fibroblast activation by sequestering specific miRNAs, including miR-484 and miR-125a-3p, thereby relieving repression of Yes1-associated transcriptional regulator (YAP1) and FYN proto-oncogene, Src family tyrosine kinase (FYN), thereby activating profibrotic signaling pathways such as STAT3 (signal transducer and activator of transcription 3) [69]. Through these circuits, RNA methylation integrates miRNA activity with non-coding RNA networks, reinforcing fibroblast activation and sustaining fibrotic remodeling in cardiovascular contexts.

Collectively, these findings suggest a multilayered regulatory framework in which m^6^A-dependent control of miRNA biogenesis, intercellular signaling, and RNA network integration may contribute to fibroblast activation and fibrotic remodeling in cardiovascular disease, while reflecting conserved mechanisms shared across diverse fibrotic conditions.

### 4.4. Cardiomyocytes

In cardiomyocytes, m^6^A-miRNA crosstalk contributes to cell fate regulation following ischemic injury, governing the balance between cell death, mitochondrial dysfunction, and regenerative responses. These regulatory effects extend across multiple processes, including pyroptosis, apoptosis, intercellular injury signaling, and cell-cycle re-entry.

m^6^A-dependent control of miRNA biogenesis represents a key mechanism that regulates cardiomyocyte death pathways under ischemic stress. In myocardial infarction, METTL14 expression is upregulated in infarcted myocardium, where it enhances m^6^A modification of pri-miR-5099 and promotes its DGCR8-dependent maturation [60]. Increased miR-5099-3p suppresses E74-like E26 transformation-specific (ETS) transcription factor 1 (ELF1), activates caspase-1 and gasdermin D (GSDMD), and ultimately drives cardiomyocyte pyroptosis. Similarly, hypoxia-induced ALKBH5-mediated m^6^A regulation of long non-coding RNA myocardial infarction-associated transcript (LncMIAT) promotes cardiomyocyte apoptosis by sponging miR-708-5p and activating p53 signaling [70]. Together, these findings indicate that m^6^A-dependent miRNA and lncRNA-miRNA pathways act as key regulators of cardiomyocyte death under ischemic stress.

Beyond cell-autonomous regulation, intercellular communication further amplifies cardiomyocyte injury signals. Hypoxia induces histone H3 lysine 4 (H3K4) methylation at the METTL3 promoter in endothelial cells, leading to increased METTL3 expression and enhanced m^6^A-dependent miR-503 biogenesis [19]. Extracellular vesicle-mediated transfer of miR-503 to cardiomyocytes suppresses sirtuin 3 (SIRT3) and peroxisome proliferator-activated receptor gamma coactivator-1β (PGC-1β), promotes mitochondrial dysfunction, and exacerbates myocardial injury. This highlights a cross-cellular regulatory axis in which m^6^A-dependent miRNA production in endothelial cells propagates injury signals to cardiomyocytes.

In contrast to these injury-promoting mechanisms, m^6^A-dependent miRNA regulation may also influence cardiomyocyte regenerative responses. METTL3 deficiency has been reported to enhance cardiac regeneration by reducing m^6^A modification of pri-miR-143, suppressing miR-143-3p maturation, and relieving repression of Yes-associated protein (YAP) and catenin delta 1 (CTNND1), thereby enabling cardiomyocyte cell-cycle re-entry [55]. This example indicates that m^6^A-miRNA circuits do not uniformly promote injury or repair, but instead exert context-dependent and disease-stage-specific effects on cardiomyocyte fate. Thus, these circuits may function as regulatory modules that influence the balance between cardiomyocyte death, mitochondrial dysfunction, and regenerative repair during cardiac remodeling.

Collectively, these findings highlight m^6^A-miRNA crosstalk as a key integrative axis that links cardiomyocyte fate regulation to cardiac remodeling outcomes. When considered together with endothelial, VSMC, and fibroblast-centered mechanisms, several common regulatory themes emerge across cardiovascular cell types. First, m^6^A-dependent control of miRNA biogenesis acts as a recurrent upstream checkpoint that shapes mature miRNA output in ECs, VSMCs, fibroblasts, and cardiomyocytes. Second, reciprocal regulation between miRNAs and the m^6^A machinery provides a feedback-like architecture through which writers, erasers, and readers can be dynamically tuned by disease-associated non-coding RNA networks. Third, m^6^A-miRNA interactions frequently converge on cell-state transitions, but their phenotypic consequences are highly cell-type specific. In ECs, these circuits mainly influence angiogenesis, barrier integrity, inflammation, apoptosis, and intercellular communication. In VSMCs, they regulate the balance between contractile stability and synthetic or remodeling-associated phenotypes. In fibroblasts and matrix-producing cells, they contribute to myofibroblast activation and extracellular matrix deposition, whereas in cardiomyocytes they affect the balance between cell death, mitochondrial dysfunction, and regenerative responses. Thus, a shared m^6^A-miRNA regulatory logic can generate distinct remodeling outcomes depending on lineage identity, disease stage, and tissue microenvironment.

## 5. Technological Advances in Epitranscriptomic-miRNA Analysis

Substantial technological advances have enabled high-resolution, cell-specific, and multidimensional interrogation of epitranscriptomic regulation. These tools increasingly support the analysis of stage-specific miRNA processing, reciprocal miRNA-m^6^A feedback, and cell-type-dependent regulatory effects.

At the single-cell level, approaches such as single-cell deamination adjacent to RNA modification targets sequencing (scDART-seq) and single-nucleus m^6^A cleavage under targets and tagmentation (m^6^A-CUT&Tag) now enable cell-resolved mapping of m^6^A modifications, revealing intercellular heterogeneity and cell-state-specific methylation landscapes [71,72]. Complementary computational frameworks, including single-cell m^6^A prediction framework (Scm^6^A), provide scalable strategies for predicting m^6^A levels from conventional scRNA-seq datasets [73]. More recent methods further extend this toolkit. For example, single-cell m^6^A sequencing (scm^6^A-seq) enables simultaneous profiling of the m^6^A methylome and transcriptome in individual cells, whereas picogram-scale methylated RNA immunoprecipitation sequencing (picoMeRIP-seq) extends m^6^A mapping to rare cells and low-input in vivo samples [74,75]. Emerging single-cell long-read strategies, such as m^6^A isoform-level single-cell RNA sequencing (m^6^A-isoSC-seq), further link m^6^A signals with isoform-resolved transcript architecture in individual cells [76]. In parallel, statistical approaches such as signature-based regression model (SigRM) support trajectory and regulatory inference from single-cell epitranscriptomic data [77]. Together, these methods provide a foundation for integrating m^6^A-marked transcripts with miRNA expression, processing, and cell-state transitions, although direct single-cell profiling of mature m^6^A-modified miRNAs remains technically challenging.

At the tissue level, spatially resolved insights can be obtained by combining m^6^A immunostaining with miRNA fluorescence in situ hybridization, allowing visualization of the colocalization of methylated RNAs and specific miRNAs within defined anatomical regions [38]. Although this approach is not equivalent to true spatial epitranscriptomics, it provides important contextual information on the spatial organization of m^6^A-miRNA interactions. Emerging spatial RNA technologies may further support future integration of RNA modification mapping with tissue architecture, but robust, transcriptome-wide spatial profiling of m^6^A-miRNA interactions remains an unmet technical goal.

Dynamic aspects of epitranscriptomic-miRNA regulation can be inferred from extracellular vesicle-mediated communication, targeted perturbation of m^6^A regulators, and time-course experimental designs. For example, endothelial-derived extracellular vesicle (EV) miRNAs generated in an m^6^A-dependent manner can modulate recipient cell function under pathological conditions, illustrating how epitranscriptomic signals propagate across cell types [19,57]. Newer technologies may help move beyond indirect inference. Nanopore direct RNA sequencing, together with tools such as SingleMod, enables single-molecule detection of m^6^A on native RNA molecules [78]. Reporter-based live-cell imaging systems such as the spatial m^6^A imaging system (SMIS) provide proof-of-concept strategies for monitoring m^6^A-dependent RNA behavior in living cells [79]. Computational tools such as miRTalk can further infer EV-derived miRNA-mediated cell–cell communication from single-cell datasets [80]. However, these approaches are not yet optimized for direct measurement of endogenous miRNA methylation dynamics in intact cardiovascular tissues.

Collectively, these technologies provide a multidimensional framework for mapping m^6^A-miRNA regulatory architecture. By integrating cell-resolved m^6^A profiling, spatial validation, native RNA sequencing, computational inference of EV-miRNA communication, and time-course studies with targeted perturbation, future studies may more directly interrogate stage-specific regulation, reciprocal interactions, and cell-type-dependent effects during cardiovascular disease progression.

## 6. Therapeutic and Translational Implications

Advances in m^6^A-miRNA crosstalk are beginning to translate mechanistic insights into potential clinical applications. By coordinating miRNA maturation, function, intercellular transfer, and feedback regulation of m^6^A machinery, this regulatory layer offers measurable disease signals and multiple potential intervention points.

### 6.1. Biomarker Development and Validation Status

Compared with conventional miRNA biomarkers [81,82], m^6^A-modified miRNAs may better capture upstream regulatory activity and cellular functional state, reflecting not only miRNA abundance but also modification-dependent regulatory context. In intracranial atherosclerosis, only m^6^A-modified miR-494-3p, rather than its unmodified counterpart, was associated with TJP1 repression and endothelial barrier dysfunction, highlighting the potential importance of modification status [38]. Circulating and vesicle-associated miRNAs further improve clinical accessibility. For example, endothelial-derived exosomal miR-302d-5p, whose maturation depends on m^6^A, has been linked to vascular calcification and aging in preclinical models [57]. In parallel, alterations in m^6^A regulators, such as METTL3 upregulation and YTHDC1 downregulation, have been associated with vascular pathology and oxidative stress responses [59,83].

However, most proposed m^6^A-miRNA biomarkers remain at the mechanistic or preclinical candidate stage. Independent validation in large, multicenter human cohorts is still limited, and few candidates have been tested across different disease stages, sample types, or patient populations. Current detection strategies include reverse transcription quantitative polymerase chain reaction (RT-qPCR) or small RNA sequencing for miRNA abundance, extracellular vesicle isolation followed by miRNA profiling for circulating vesicle-associated miRNAs, immunoprecipitation-based approaches such as methylated RNA immunoprecipitation quantitative polymerase chain reaction (MeRIP-qPCR) for m^6^A-modified RNAs, and targeted assays for selected modified miRNAs. Each approach has important limitations. RT-qPCR and sequencing are sensitive for abundance measurement but do not directly resolve modification status. Immunoprecipitation-based assays can detect methylated RNA species but may suffer from antibody-dependent variability, limited site resolution, and relatively high input requirements. Extracellular vesicle-based assays are attractive for liquid biopsy but remain sensitive to differences in sample processing, vesicle isolation, normalization strategy, and inter-laboratory reproducibility. Therefore, clinical implementation will require standardized protocols for sample collection, RNA extraction, EV isolation, modification detection, normalization, and independent cohort validation. The translational status of representative m^6^A-miRNA biomarkers is summarized in Table 3.

### 6.2. Therapeutic Targeting Strategies and Evidence Levels

Therapeutic targeting of m^6^A-miRNA crosstalk is emerging as a potential strategy to modulate cardiovascular remodeling, although the current evidence is predominantly preclinical. Several experimental studies have shown that manipulating components of the m^6^A machinery can alter miRNA maturation or function and thereby influence disease-relevant vascular phenotypes. For example, METTL3 silencing was reported to stabilize atherosclerotic plaques by regulating vascular smooth muscle cell phenotypic transformation [59], while METTL3-driven m^6^A modification of the miR-143/145-KLF4 circuit was shown to orchestrate pulmonary artery smooth muscle cell phenotypic switching in pulmonary hypertension models [36]. Similarly, modulation of m^6^A-associated circRNA/miRNA axes or miRNA-dependent regulation of m^6^A writers has been linked to vascular neointimal hyperplasia and calcification [45,49]. These findings support the therapeutic potential of targeting m^6^A-miRNA regulatory nodes, but they remain largely based on cell-based assays and animal models rather than human interventional studies.

In addition to targeting individual m^6^A regulators or miRNAs, circuit-level intervention may provide a network-based strategy when reciprocal regulation between non-coding RNAs and the m^6^A machinery sustains disease-relevant vascular states. As discussed above, the circ_0103896/miR-432-5p/FTO axis in intracranial aneurysm provides a representative example of a positive circRNA-miRNA-eraser feedback loop [52]. Importantly, this circuit was experimentally perturbed in vivo: circ_0103896 overexpression alleviated aneurysm severity, whereas miR-432-5p overexpression or pharmacological inhibition of FTO with FB23-2 attenuated this protective effect. Thus, this study provides preclinical proof-of-concept evidence that an epitranscriptomic-miRNA feedback circuit can be manipulated at the circuit level to influence vascular remodeling phenotypes. Nevertheless, such evidence remains confined to experimental models, and no clinical trials have yet tested therapeutic interventions specifically designed to modulate m^6^A-miRNA regulatory circuits in cardiovascular remodeling. These findings indicate that future circuit-level strategies should define disease-stage-specific intervention windows, distinguish protective from pathogenic regulatory loops, and evaluate delivery feasibility, durability, and safety before clinical translation.

The broader field of epitranscriptomic therapeutics is beginning to enter clinical development, but this progress is currently concentrated outside cardiovascular indications. For example, the METTL3 inhibitor STC-15 is being evaluated in a Phase 1 clinical study in patients with advanced malignancies (ClinicalTrials.gov ID: NCT05584111), illustrating that pharmacological inhibition of m^6^A writing has begun to enter clinical development outside cardiovascular indications. These developments support the clinical tractability of pharmacologically targeting RNA-modifying enzymes. However, they do not yet establish therapeutic feasibility for cardiovascular remodeling, where long-term disease modification, tissue-selective delivery, vascular and myocardial safety, and preservation of physiological RNA methylation programs are particularly important. Thus, translation of m^6^A-miRNA-based strategies for cardiovascular disease remains at an early preclinical stage and will require cardiovascular-specific validation before clinical application.

### 6.3. Delivery and Safety Considerations

Clinical translation of m^6^A-miRNA-based strategies remains constrained by both delivery barriers and safety concerns. Because the m^6^A machinery regulates broad classes of transcripts, including mRNAs, lncRNAs, circRNAs, primary miRNAs (pri-miRNAs), and precursor miRNAs (pre-miRNAs), nonselective or sustained modulation of core writers, erasers, or readers may induce transcriptome-wide and epitranscriptome-wide off-target effects. Such effects could disrupt physiological RNA methylation programs that maintain vascular homeostasis, immune regulation, and tissue repair. This concern is particularly relevant because global deletion of key m^6^A components, such as METTL3, METTL14, or WTAP, causes early embryonic lethality in mice, and cell-specific perturbation of m^6^A regulators affects the maintenance of multiple organs and immune functions [84]. Therefore, future therapeutic strategies should avoid long-term systemic perturbation of essential m^6^A regulators whenever possible, and instead prioritize cell-type-selective, temporally controlled, and reversible approaches that target disease-enriched regulatory nodes. Safety evaluation should include not only target-gene readouts but also transcriptome-wide RNA expression profiling, m^6^A mapping, miRNA profiling, and assessment of unintended effects on normal vascular and immune cell functions.

Delivery platforms are also central to both efficacy and safety, especially for cardiovascular applications. Engineered extracellular vesicles, liposome-EV hybrids, and targeted nanoparticles can improve nucleic acid stability, cargo protection, and cell-type specificity in experimental settings [85,86,87]. In cardiovascular disease, targeted delivery of miRNA-modulating antisense oligonucleotides has been proposed to improve tissue selectivity and reduce systemic exposure, but clinical translation remains limited by delivery efficiency, biodistribution, and safety concerns [88]. Oligonucleotide cargos, including small interfering RNAs (siRNAs), antisense oligonucleotides (ASOs), and miRNA-targeting oligonucleotides, may cause sequence-dependent off-target repression or innate immune activation, including type I interferon and inflammatory cytokine responses; these effects can be amplified or attenuated by carrier composition, chemical modification, and route of administration [89,90]. For example, cationic lipid-mediated systemic delivery of immunostimulatory siRNA induced type I interferons and inflammatory cytokines, whereas atelocollagen-mediated delivery avoided these immunostimulatory effects in animal models and human peripheral blood mononuclear cells [91]. In parallel, nanocarriers introduced into the vascular system can interact with endothelial cells, influence endothelial stress responses, and, at high concentrations or prolonged exposure, induce oxidative stress, membrane damage, apoptosis, or inflammatory responses [92]. Therefore, preclinical development of m^6^A-miRNA-based therapeutics should assess biodistribution, dose-dependent efficacy and toxicity, inflammatory and hematological responses, liver and kidney function, repeat-dose tolerability, and the durability and reversibility of the intended m^6^A-miRNA perturbation before clinical translation [89,92,93].

## 7. Conclusions and Outlook

Currently, m^6^A RNA methylation-miRNA crosstalk is emerging as a unifying regulatory layer that may link dynamic environmental cues to stable gene expression programs during cardiovascular remodeling. Rather than functioning as independent pathways, m^6^A RNA methylation and miRNAs interact across multiple regulatory dimensions, including stage-specific checkpoints, reciprocal feedback loops, and lineage-specific circuits across the vessel wall and myocardium. Together, these interconnected regulatory levels provide a conceptual framework for understanding how transient pathological stimuli may contribute to durable remodeling phenotypes.

However, the current state of the field remains largely mechanistic and preclinical. Several fundamental questions are still unresolved, including the cell-type specificity, context dependence, and disease-stage dependence of m^6^A-dependent miRNA regulation. In particular, it remains unclear how distinct combinations of m^6^A marks and miRNA activities are decoded into cell-specific functional outputs, and how the plasticity of these regulatory programs changes during disease progression. In addition, current technologies still have limited capacity to directly resolve RNA methylation dynamics with sufficient spatial, temporal, and molecular resolution, which constrains our ability to track how these regulatory interactions evolve in vivo.

A critical limitation of the current literature is that many reported m^6^A-miRNA axes have been characterized in single disease models or specific cell types, with limited cross-validation across cardiovascular contexts. Therefore, mechanisms identified in pulmonary hypertension, atherosclerosis, aneurysm formation, myocardial infarction, or fibrosis should not be assumed to operate equivalently across all forms of cardiovascular remodeling. Discrepancies may also arise between in vitro and in vivo systems. Cell culture models allow controlled perturbation of m^6^A regulators or miRNAs, but they may not fully recapitulate hemodynamic stress, multicellular interactions, inflammatory cues, metabolic remodeling, or extracellular matrix changes present in diseased tissues. Conversely, in vivo models provide physiological context, but it can be difficult to assign phenotypes specifically to m^6^A-dependent miRNA regulation rather than broader effects on RNA splicing, stability, translation, or decay. Methodologically, some studies infer m^6^A-dependent regulation from changes in RNA abundance, immunoprecipitation enrichment, or global manipulation of writers, erasers, and readers, without directly measuring site-specific modification, RNA half-life, or miRNA processing kinetics. Disease stage, species, cell source, delivery method, sequencing depth, antibody specificity, and normalization strategy may further contribute to variability across studies.

Future integration of multi-omics datasets, systems biology approaches, and machine learning or artificial intelligence methods may facilitate the identification, modeling, and predictive analysis of m^6^A-miRNA regulatory networks across cardiovascular contexts.

Looking forward, future studies should move beyond single-axis validation toward integrated, cell-resolved, and temporally controlled analyses of m^6^A-miRNA regulatory networks. Key priorities include improving detection sensitivity, developing spatial and single-cell epitranscriptomic approaches, defining protective versus pathogenic circuits, and mapping disease-stage-specific remodeling phenotypes. These efforts should combine orthogonal validation methods, cell-type-specific perturbation, time-course designs, and in vivo functional assays to distinguish causal m^6^A-miRNA mechanisms from associative regulatory signatures. From a translational perspective, further progress will require precise cell-type-specific delivery, careful assessment of off-target effects, and rigorous evaluation of long-term safety before m^6^A-miRNA-based strategies can be considered for clinical application. Beyond m^6^A, other RNA modifications, such as m^5^C, m^1^A, and pseudouridylation, may also influence non-coding RNA biology. However, their direct links to miRNA regulation in cardiovascular remodeling remain less well defined and warrant future investigation.

Ultimately, clearer delineation of this regulatory architecture may provide a foundation for next-generation precision strategies that target dynamic RNA regulatory networks rather than individual molecular components. Such a shift could move therapeutic thinking in cardiovascular disease from single-target intervention toward context-aware and systems-level modulation of pathological remodeling.

## Figures and Tables

**Table 1:** m^6^A-miRNA circuits in cardiovascular remodeling.

miRNA(s)	m^6^A Machinery	Affected Cell Type	Disease Context	Model/Experimental System	Effects of m^6^A Regulation on miRNAs	Biological Outcome	Reference
let-7e-5p, miR-18a-5p	METTL3	ECs	Ischemic injury	Limb ischemia/MI mice; EC assays	Enhances pri-miRNA processing	Promotes angiogenesis	[54]
miR-143-3p	METTL3	CMs	Myocardial infarction	METTL3-deficient LAD-MI mice; neonatal CMs	Enhances pri-miRNA processing	Promotes cardiomyocyte proliferation and heart repair	[55]
miR-143-3p, miR-145-5p	METTL3, hnRNPA2B1	PASMCs	Pulmonary hypertension	SMC-*Mettl3* KO hypoxia-PH mice; PASMC assays	Enhances pri-miRNA processing	Maintains PASMC contractile phenotype	[36]
miR-185	hnRNPA2B1	VSMCs → ECs	Restenosis after vascular reconstruction	Carotid injury rats; PDGF-VSMCs/ECs	Controls EV miRNA sorting	Impairs EC function	[44]
miR-19a	METTL14	ECs	Atherosclerosis	ASVECs; human AS plaques	Enhances pri-miRNA processing	Promotes the proliferation and invasion of endothelial cells	[35]
miR-20b	METTL3	ECs	Hypoxia/reoxygenation injury	H/R-HUVECs; propofol treatment	Enhances pri-miRNA processing	Inhibits excessive autophagy	[56]
miR-302d-5p	WTAP, hnRNPA2B1	ECs → VSMCs	Chronic kidney disease-induced vascular calcification and aging	5/6 nephrectomy CKD mice; MT-EC EVs/VSMCs	Enhances pri-miRNA processing	Inhibits VSMC calcification and senescence	[57]
miR-34a	METTL3	VSMCs	Abdominal aortic aneurysm	Ang II-AAA mice; VSMC assays	Enhances pri-miRNA processing	Exacerbates vascular remodeling	[58]
miR-375-3p	METTL3	VSMCs	Atherosclerosis	*ApoE*^−/−^ HFD mice; ox-LDL-MOVAS	Enhances pri-miRNA processing	Modulates VSMC phenotypic switching and exacerbates atherosclerosis	[59]
miR-503	METTL3	ECs → CMs	Acute myocardial infarction	LAD-AMI mice; hypoxic ECs/CMs	Enhances pri-miRNA processing; controls EV miRNA sorting	Induces cardiomyocyte mitochondrial dysfunction and exacerbates cardiac injury	[19]
miR-5099-3p	METTL14	CMs	Myocardial infarction	LAD-MI mice; hypoxic neonatal CMs	Enhances pri-miRNA processing	Promotes cardiomyocyte pyroptosis	[60]
miR-636	FTO	CFs	Myocardial fibrosis	LAD-AMI mice; Ang II-CFs	Modifies miRNA targeting	Suppresses myocardial fibrosis	[18]
miR-7	ALKBH5	ECs	Atherosclerosis	TNFα-HUVECs; ALKBH5 overexpression	Inhibits pri-miRNA processing	Protects endothelial cells from apoptosis; sustains Bcl-2 expression	[61]
miR-99a	hnRNPA2B1	ECs → CD4+ T cells	Inflammatory dilated cardiomyopathy	MyHCα-iDCM mice; LIPUS-treated EC/CD4+ T cells	Controls EV miRNA sorting	Alleviates immune-inflammatory responses and regulates myocardial remodeling	[43]
miR-103-3p	hnRNPA2B1	EPCs → macrophages	Acute respiratory distress syndrome-associated endothelial inflammation	LPS-ARDS mice; EPC-EVs/RAW264.7	Controls EV miRNA sorting	Promotes M2 macrophage polarization and alleviates acute lung injury	[62]
miR-34a-5p	METTL14	ECs	Preeclampsia-associated endothelial injury	TNFα-HUVECs; METTL14/FOXP1 knockdown	Enhances pri-miRNA processing	Promotes vascular endothelial cell inflammation and injury	[63]

Note: Arrows indicate the major direction of intercellular miRNA transfer or functional influence. The “Model/experimental system” column summarizes the principal disease model, sample source, stimulation condition, or key intervention. Disease contexts outside classical cardiovascular disorders are included only when mechanistically relevant. Most listed axes are supported by mechanistic or preclinical evidence; potential therapeutic relevance is discussed in Section 6.

**Table 2:** miRNA regulation of RNA methylation machinery in cardiovascular remodeling.

miRNA	Targeted mRNA	Biological Function	Disease Context	Reference
miR-17-3p	YTHDF2	Exacerbates endothelial dysfunction and vascular remodeling	Pulmonary hypertension	[48]
miR-19b-3p	YTHDF1	Suppresses vascular endothelial ferroptosis and apoptosis	Pregnancy-induced hypertension	[50]
miR-33a-5p	METTL3	Suppresses VSMC calcification	Atherosclerosis	[45]
miR-4729	METTL14	Inhibits angiogenesis	Hemorrhoidal vascular hyperplasia	[46]
miR-636	YTHDF2	Suppresses VSMC autophagy	Neointimal hyperplasia	[49]
miR-let7	IGF2BP1	Suppresses inflammatory cell infiltration and promotes M2 polarization	Atherosclerosis	[51]

**Table 3:** Candidate m^6^A-miRNA biomarkers in cardiovascular remodeling.

Biomarker Candidate	Disease Context	Evidence Level	Detection Approach	Current Limitation	Reference
m^6^A-modified miR-494-3p	Intracranial atherosclerosis	Human association and mechanistic evidence	Modified-miRNA detection with functional assays	Limited independent cohort validation; assay standardization needed	[38]
Exosomal miR-302d-5p	Vascular calcification and aging	Preclinical and intercellular-transfer evidence	EV isolation and miRNA quantification	EV isolation, normalization, and reproducibility need standardization	[57]
METTL3-related miRNA maturation signatures	Pulmonary hypertension, atherosclerosis, myocardial infarction	Mechanistic and preclinical evidence	RT-qPCR, small RNA profiling, and m^6^A-related assays	Disease specificity and predictive value remain unclear	[36,59]
m^6^A regulator expression signatures	Vascular pathology and oxidative-stress remodeling	Association-level or mechanistic evidence	qPCR, immunoblotting, immunostaining, or transcriptomics	Not specific to m^6^A-miRNA crosstalk; requires functional and cohort validation	[59,83]

Note: Most candidates remain at mechanistic, preclinical, or association-based stages; independent cohort validation and standardized detection workflows are required before clinical application.

## Data Availability

No new data were created or analyzed in this study. Data sharing is not applicable to this article.

## Abbreviations

The following abbreviations are used in this manuscript:

AAA	abdominal aortic aneurysm
AAV9	adeno-associated virus serotype 9
AGO2	Argonaute 2
ALKBH5	AlkB homolog 5
AMI	acute myocardial infarction
Ang II	angiotensin II
ApoE	apolipoprotein E
ARDS	acute respiratory distress syndrome
AS	atherosclerosis
ASO(s)	antisense oligonucleotide(s)
ASVEC(s)	atherosclerotic vascular endothelial cell(s)
Bcl-2	B-cell lymphoma 2
ceRNA	competing endogenous RNA
CF(s)	cardiac fibroblast(s)
circRNA	circular RNA
CKD	chronic kidney disease
CM(s)	cardiomyocyte(s)
CTNND1	catenin delta 1
DGCR8	DiGeorge syndrome critical region 8
DKK2	dickkopf Wnt signaling pathway inhibitor 2
EC(s)	endothelial cell(s)
ELF1	E74-like ETS transcription factor 1
EPC(s)	endothelial progenitor cell(s)
ETS	E26 transformation-specific
EV(s)	extracellular vesicle(s)
FOXM1	forkhead box M1
FOXP1	forkhead box protein P1
FTO	fat mass and obesity-associated protein
FYN	FYN proto-oncogene, Src family tyrosine kinase
GSDMD	gasdermin D
H/R	hypoxia/reoxygenation
H3K4	histone H3 lysine 4
HFD	high-fat diet
hnRNPA2B1	heterogeneous nuclear ribonucleoprotein A2/B1
HUVEC(s)	human umbilical vein endothelial cell(s)
iDCM	inflammatory dilated cardiomyopathy
IGF2BP1	insulin-like growth factor 2 mRNA-binding protein 1
IGF2BP2	insulin-like growth factor 2 mRNA-binding protein 2
KLF4	Krüppel-like factor 4
KO	knockout
LAD	left anterior descending coronary artery
LINC01116	long intergenic non-protein coding RNA 1116
LIPUS	low-intensity pulsed ultrasound
LncMIAT	long non-coding RNA myocardial infarction-associated transcript
lncRNA	long non-coding RNA
LPS	lipopolysaccharide
m^6^A	N6-methyladenosine
m^6^A-CUT&Tag	m^6^A cleavage under targets and tagmentation
m^6^A-isoSC-seq	m^6^A isoform-level single-cell RNA sequencing
MALAT1	metastasis-associated lung adenocarcinoma transcript 1
MeRIP-qPCR	methylated RNA immunoprecipitation quantitative polymerase chain reaction
METTL14	methyltransferase-like 14
METTL3	methyltransferase-like 3
MI	myocardial infarction
miRNA	microRNA
miRTalk	miRNA-mediated cell–cell communication inference tool
MOVAS	mouse aortic vascular smooth muscle cell line
mRNA	messenger RNA
MT	melatonin
mTOR	mechanistic target of rapamycin
MyHCα	alpha-myosin heavy chain
ox-LDL	oxidized low-density lipoprotein
PASMC(s)	pulmonary artery smooth muscle cell(s)
PDGF	platelet-derived growth factor
PDK1	3-phosphoinositide-dependent protein kinase 1
PGC-1β	peroxisome proliferator-activated receptor gamma coactivator-1β
PH	pulmonary hypertension
picoMeRIP-seq	picogram-scale methylated RNA immunoprecipitation sequencing
pre-miRNA	precursor microRNA
pri-miRNA	primary microRNA
PTK2	protein tyrosine kinase 2
RAW264.7	murine macrophage cell line
RBP	RNA-binding protein
RISC	RNA-induced silencing complex
RT-qPCR	reverse transcription quantitative polymerase chain reaction
scDART-seq	single-cell deamination adjacent to RNA modification targets sequencing
Scm^6^A	single-cell m^6^A prediction framework
scm^6^A-seq	single-cell m^6^A sequencing
SigRM	signature-based regression model
SingleMod	single-molecule RNA modification detection tool
siRNA(s)	small interfering RNA(s)
SIRT1	sirtuin 1
SIRT3	sirtuin 3
SMC(s)	smooth muscle cell(s)
SMIS	spatial m6A imaging system
TIE1	tyrosine kinase with immunoglobulin-like and EGF-like domains 1
TJP1	tight junction protein 1
TNFα	tumor necrosis factor alpha
ULK1	unc-51-like autophagy activating kinase 1
VEGFA	vascular endothelial growth factor A
VSMC(s)	vascular smooth muscle cell(s)
WTAP	WT1-associated protein
YAP	Yes-associated protein
YAP1	Yes1-associated transcriptional regulator
YTHDC	YTH domain-containing protein family
YTHDC1	YTH domain-containing protein 1
YTHDC2	YTH domain-containing protein 2
YTHDF	YTH N6-methyladenosine RNA-binding protein family
YTHDF1	YTH N6-methyladenosine RNA-binding protein 1
YTHDF2	YTH N6-methyladenosine RNA-binding protein 2
